# Thalassemia in Bangladesh: progress, challenges, and a strategic blueprint for prevention

**DOI:** 10.1186/s13023-025-03744-x

**Published:** 2025-07-10

**Authors:** Mohammad Sorowar Hossain, Farhin Islam, Selina Akhter, Abdullah Al Mossabbir

**Affiliations:** 1 Department of Emerging and Neglected Diseases, Biomedical Research Foundation, Dhaka, Bangladesh; 2 School of Environment and Life Sciences, Independent University, Bangladesh, Dhaka, Bangladesh; 3 Bangladesh Institute of Development Studies (BIDS), Dhaka, Bangladesh; 4 Department of Obstetrics and Gynecology, Gopalganj Medical College, Gopalganj, Bangladesh; 5 BMT Unit, Department of Hematology, Dhaka Medical College Hospital, Dhaka, Bangladesh

**Keywords:** Thalassemia, Bangladesh, Prevention, Management, Progress, Challenges, Update, Patients

## Abstract

Thalassemia, a preventable hereditary disorder causing defective hemoglobin production, presents a growing public health concern in Bangladesh, a country situated within the Global Thalassemia Belt. This review systematically synthesizes all available data on the prevalence, clinical characteristics, management challenges, and socio-economic implications of thalassemia in Bangladesh, offering a blueprint for a prevention strategy tailored to its unique socio-economic and cultural context. The review highlights a high carrier prevalence of 10.9–13.3%, translating to approximately 17–22 million carriers, with significant regional and ethnic variations, including a disproportionately high E-trait prevalence. Most transfusion-dependent thalassemia cases in Bangladesh are Hb E-Beta Thalassemia. With proper care, many patients can avoid regular transfusions. However, clinical management remains inadequate due to insufficient healthcare infrastructure, poor access to safe blood transfusions, limited iron chelation therapy, and a lack of multidisciplinary care. These gaps are compounded by socio-economic barriers, with most patients bearing 74% of treatment costs out-of-pocket, leaving many deprived of adequate care. Building on successful international prevention models from countries such as Cyprus, Iran, and Italy, the paper proposes a high school-centric intervention strategy for Bangladesh. This includes awareness campaigns, carrier screening, and genetic counseling targeted at adolescents, coupled with the integration of thalassemia education into school curricula. Additionally, it calls for the establishment of district-level treatment centers, a national patient registry, and policies to subsidize treatment costs. Despite the cultural and logistical challenges, the review underscores the urgent need to prioritize awareness, early diagnosis, and prevention over treatment. It emphasizes that thalassemia is theoretically 100% preventable through premarital screening and prenatal diagnosis if implemented with religious and cultural sensitivity. By providing a roadmap for a comprehensive thalassemia prevention and management strategy, this review aims to guide policymakers and stakeholders while improving the quality of life for existing patients.

**Supplementary Information:**

The online version contains supplementary material available at 10.1186/s13023-025-03744-x.

## Introduction

Thalassemia is a potentially preventable hereditary disorder caused by the defective gene that produces defective hemoglobin resulting in anemia. Depending on the type and severity of the disease, it causes various health problems. Thalassemia is mainly of two types. When the genes associated with the alpha globin protein are absent or mutated, it is called Alpha thalassemia. When a similar gene defect affects the production of beta globin protein, it is called Beta-thalassemia. According to the clinical severity of the disease, there are two types of thalassemia– thalassemia carrier or thalassemia trait, and thalassemia syndrome or thalassemia disease. Heterozygotes of alpha and beta thalassemia are called thalassemia carriers. They are not patients. Thalassemia carriers usually do not need any medication or treatment and can live like healthy people, even showing no symptoms in most cases.

On the other hand, homozygotes and compound heterozygotes of alpha or beta thalassemia genes give rise to various thalassemia syndromes or thalassemia diseases. Currently, based on their transfusion requirement, these thalassemia syndromes can be classified into two groups: (1) Transfusion Dependent Thalassemia (TDT) and (2) Non-Transfusion Dependent Thalassemia (NTDT).

Patients with Transfusion-dependent thalassemia (TDT) require regular treatment. If both parents are carriers of thalassemia, there is a 25% chance that the disease will be passed on to the child in each pregnancy. This category includes patients with beta-thalassemia major, severe Hb E-Beta thalassemia and transfusion-dependent alpha thalassemias. People with TDT suffer from severe life-threatening anemia. As a result, patients suffer from stunted physical development and several other serious health problems, which can lead to premature death if left untreated. They require life-long blood transfusions, iron-chelation medications, and multidisciplinary care for survival. Patients with Non-transfusion-dependent Thalassemia (NTDT) do not require lifelong regular transfusions for survival, although may require occasional or even frequent transfusions in certain clinical settings and for defined period of time [1, 2]. Thalassemia is widely prevalent in Southeast Asia, the Indian subcontinent, the Mediterranean, Central Asia, the Middle East and West Africa [3], which is geographically known as the Global Thalassemia Belt. This is because thalassemia outbreaks have historically been associated with malaria outbreaks (Malaria Hypothesis by J. B. S. Haldane, 1948). However, thalassemia has spread to other regions such as Europe and North America due to migration [4]. Thalassemia has become a health concern worldwide due to its high prevalence, inadequacy of treatment, imperative of prevention, and lack of cure.

Fifty-six thousand children are born with thalassemia every year in the world [5]. International Thalassemia Day is celebrated worldwide on May 8 every year to raise awareness about the disease. In 2006, the World Health Organization declared thalassemia a global public health problem [6]. Despite Bangladesh being one of the hotspots in the world for thalassemia, thalassemia has not yet been sufficiently recognized as a public health problem in the country. Ministry of Health has announced some initiatives with the intention of preventing this silent killer and creating a patient support system but they have not been implemented yet. Only due to a lack of awareness among the masses, the disease is slowly spreading like a silent epidemic across the country.

Thalassemia is so neglected that international standard research on the disease started in Bangladesh just a few years ago. In 2017, a non-profit charitable organization, Biomedical Research Foundation (BRF) and Bangladesh Thalassemia Foundation (BTF) jointly published the first comprehensive report on the overall status of the disease in the internationally renowned journal [7]. The objective of this study is to review all the data available so far about thalassemia in Bangladesh. Based on the knowledge already obtained, this study aims to shed light on a correct and effective strategy to prevent thalassemia in the socio-economic context of Bangladesh by learning from other countries.

## Methodology

### Search strategy

**Fig. 1:**
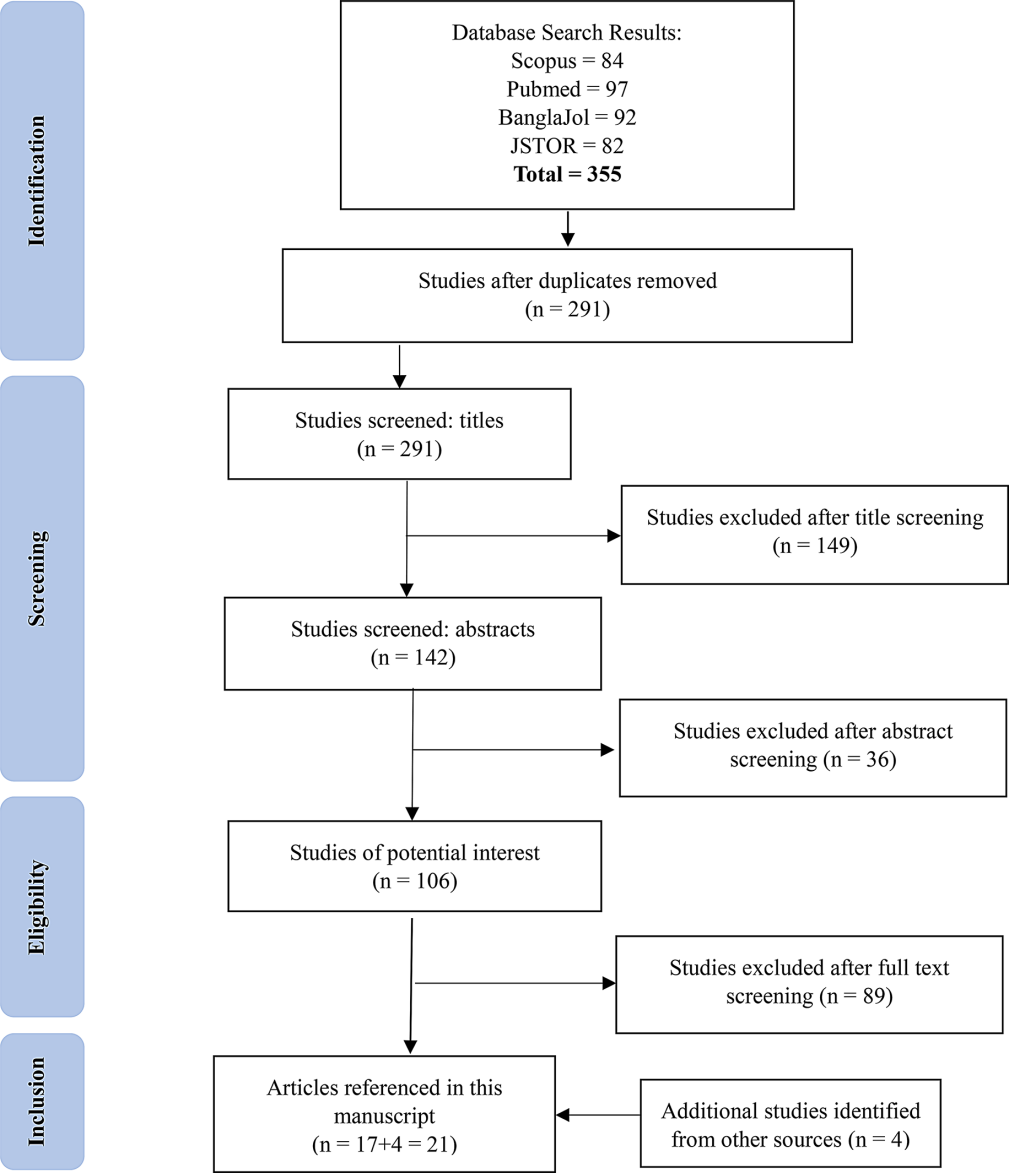
Systematic search for journal articles describing Thalassemia in Bangladesh

We conducted a systematic narrative literature review on the current status of epidemiology, clinical spectrum, treatment and complications, and socioeconomic factors related to thalassemia in Bangladesh. However, we did not register any protocol for this review. For this purpose, we searched Scopus, PubMed, and JSTOR on 5 December 2024, for studies published in English describing the diagnosis, treatment, prevalence, mental health, quality of life, awareness, prevention and other socioeconomic issues regarding thalassemia in Bangladesh, using the search terms “Thalassemia” or “Thalassaemia” and “Bangladesh”. Bangladeshi articles were searched in the BanglaJol database (an exclusive database of Bangladeshi Journal) with the keywords “Thalassemia” and “Prevention”, “Treatment”, “Diagnosis”, “Prevalence”, “Iron”, “Blood”, “Hemoglobin”, “Trait”, “Knowledge”, “Anemia” etc. Figure 1 depicts the systematic search of published articles in all these sources and Additional file 1 presents the process of step-by-step analysis of the search results.

## Eligibility criteria

In this study, specific criteria were used to select relevant articles for analysis. The inclusion criteria were: (i) articles describing the prevalence and epidemiology, diagnosis and treatment, clinical manifestations, genetic mutations, preventive measures, socioeconomic factors, mental health and knowledge and awareness regarding Thalassemia in Bangladesh, (ii) articles published in English and (iii) peer-reviewed original articles, review articles, case studies etc. The exclusion criteria were: (i) non-peer-reviewed articles (such as editorials, comments, and conference presentations), (ii) articles published in other languages, (iii) articles based on other countries or group of countries and (iv) articles focused on another related disease.

## Selection of studies and data extraction

The final search results were combined from all sources, inserted into Excel and duplicates were removed. Two reviewers (MSH and FI) screened the title and abstract. Articles of potential interest were defined based on themes (Diagnosis, Treatment, Prevalence, Socioeconomic, Overview, Mental Health or Quality of Life, Awareness and Prevention) and types (Clinical vs. Non-clinical). Full texts of the primarily selected articles were then screened thoroughly. Any discrepancies found during the selection of studies were resolved through discussion and consensus of the two reviewers which were then evaluated by a third reviewer (AAM).

## Prevalence of thalassemia in Bangladesh

Although Bangladesh is a small lower-middle-income country, it is densely populated with about 170 million people [8]. Before the publication of our first study (2017) which is the first comprehensive paper on thalassemia in Bangladesh [7], there was only one small study (2005) on the prevalence of thalassemia in Bangladesh [9], but the carrier screening system was different in that study (they did not use the Hemoglobin Electrophoresis test). The latest figure from the National Thalassemia Survey 2024, based on data from individuals aged 14 to 35 across Bangladesh, reveals that the overall prevalence of thalassemia carriers is 11.4% [10]. Based on all studies so far conducted (excluding tribal population) since the publication of the first comprehensive paper (2017) in Bangladesh [7] (including studies on college/ university students, garment workers, and nationwide), the total thalassemia carrier prevalence is 10.9–13.3% which translates to around 17–22 million carriers. The prevalence of E-trait is found to be around 8–10%, while the prevalence of Beta-trait is around 2–3%.

There are also regional variations in the incidence of thalassemia in Bangladesh. According to the National Thalassemia Survey 2024 report, the highest prevalence of thalassemia carrier is in Rangpur division (27.7%) followed by Rajshahi Division (11.3%) and Chattogram Division (11.2%) and the lowest prevalence is in Sylhet Division (4.8%) [10]. On the other hand, the prevalence of trait was highest among the indigenous community at Chittagong hill tracts (~ 40%) [11]. While the prevalence of E-trait among the indigenous community is significantly higher compared to the general population, the prevalence of Beta-trait is similar [11–13]. Another study conducted among 1877 young individuals of marriageable age (18–35 years) also found a similar variation by type and region [12].

It has been estimated that 5–7% of the world’s population carries hemoglobinopathy [5]. The prevalence of E-trait is disproportionately higher from Beta-trait in Bangladesh and this experience is significantly different from other countries. In the neighboring country India, the prevalence of both E-trait and Beta-trait is around 2–3% but in West Bengal, the prevalence of Beta-trait is significantly higher. Sri Lanka and Kuwait have the same kind of experience. We do not have information on the prevalence of E-trait in Pakistan, Iran, Turkey and Saudi Arabia. In Pakistan, Sri Lanka and Iran, the prevalence of Beta-trait is around 4–5%. In Kuwait, Turkey and Saudi Arabia, it is around 2%. Thailand is a special case (somewhat similar to Bangladesh) where the prevalence of E-trait is very high compared to that of Beta-trait (Table 1).

**Table 1:** Prevalence of thalassemia in Bangladesh and other countries

Country	Period	Sample size	E-trait (%)	Beta-trait (%)	Combined	Population	Source
Bangladesh	2005	735	6.1	4.1	10.2	School children	[9]
	2010-11	202	35.2	N/A	N/A	Tribal population (Marma and Khyang)	[63]
	2018-19	114	38.6	1.8	40.4	Indigenous university students	[11]
	2018	989	10.2/10.8	3.1/1.8	13.3/12.6	University students/ Garment workers	[13]
	2018-19	1877	8.68	2.24	10.92	Young unmarried adults from all 8 administrative divisions across Bangladesh	[12]
	2018–2019	1439	10.8	2.4	13.2	College & university students	Personal communication
	2024	8680 households			11.4	Nationally representative survey covering individuals aged 14–35 years	[10]
India (West Bengal)	2010-20	287,258	2.77	7.23	10	Rural areas of West Bengal (West Midnapore and Jhargram districts located in the southern part)	[64]
India	2000-05	56,780	3.63	2.78	6.41	College students and pregnant women from different states	[65]
	1978–2022	703,615 (Meta-analysis)	N/A	3.74	N/A	General population (students, women, community,	[66]
Pakistan	2010	181	N/A	5.5	N/A	General population (Subjects were self-recruited on thalassemia day by advertisement through media)	[67]
	2016-17	2279	N/A	4.25	N/A	Healthy individuals who came from all over Pakistan for central medical board for selection in Pakistan Air Force	[68]
Sri Lanka	2017-18	1821	1.2	5.7	6.9	School children aged 14–17 years in Kurunegala district	[69]
Thailand	2012	350	17.1	0.6	17.7	350 cord blood specimens collected consecutively at Maternal and Child Hospital, Regional Health Promotion Center 7, Khon Kaen, Thailand	[70]
Vietnam	2020-21	10,112	N/A	2.24	N/A	Pregnant women and their husbands visiting the Vietnam National Hospital of Obstetrics and Gynecology	[71]
Saudi Arabia	2004-09	1,572,140	N/A	1.79	N/A	All couples with marriage proposals	[72,73]
Iran	1989-95	N/A	N/A	4	N/A	Countrywide	[74]
	2014-17	17,581	N/A	5.6	N/A	Khuzestan Province, Southwest Iran	[75]
Turkey	1971	1000	N/A	2.1	N/A	900 from healthy subjects in the Turkish Army and the Medical School and 100 cord blood samples from City Maternity Hospital, Ankara	[76,77]
Kuwait	2009-20	275,819	0.053	2.12	2.173	Adult population screened as part of the National Premarital Screening Program	[78]

Regarding thalassemia disease, we extrapolated that nearly 70,000 patients are suffering from various thalassemia syndromes in Bangladesh. In addition, around 10,000 children with thalassemia are being added each year with more than 2000 cases with TDT [7, 12, 14]. Hb E-Beta thalassemia is more common in Bangladesh than Beta thalassemia major (Table 2). Data from published papers and different thalassemia care centers suggests that more than 70% of patients in Bangladesh are Hb E-Beta Thalassemia, less than 30% are Beta thalassemia major and other hemoglobinopathy constitutes less than 5%. Hb E-Beta thalassemia is also common in India (particularly West Bengal) [15] and some other countries from Southeast Asia and the Indian subcontinent like Thailand, Indonesia, and Sri Lanka [16].

**Table 2:** Clinical characteristics of thalassemia syndromes in Bangladesh

Parameter	Sample	Year/Period	Sample size	%	Study setting	Source
Thalassemia Patient	Among thalassemic children	2018	365	Beta thalassemia major: 29.1 Hb E-Beta thalassemia: 70.1	A dedicated thalassemia hospital located in Dhaka (BTSH)	[14]
	Attending thalassemia patients	2017	1178	Beta thalassemia major: 14.69 Hb E-Beta thalassemia: 77.25	A dedicated thalassemia hospital located in Dhaka (BTF)	[7]
	Among thalassemia patients	2020	42	Beta thalassemia major: 38.1 Hb E-Beta thalassemia: 61.9	Registered thalassemia patients in community setting in Jamalpur	[79]
Transfusion dependent thalassemia	Attending thalassemia patients	2017	1063	66.98	A dedicated thalassemia hospital located in Dhaka (BTF)	[7]
Non-transfusion dependent thalassemia				24.36		
The frequency of blood transfusion per month	Among thalassemic children (having beta thalassemia major or Hb E beta thalassemia)	2018	365	Once: 47.1 Twice: 42.2 3 times: 8.2 > 3 times: 2.5	A dedicated thalassemia hospital located in Dhaka (BTSH)	[14]
Iron chelating drug	Attending patients with multiple blood transfusions	2017	972	43% Deferiprone: 481/972 Deferasirox: 199/972 Desferal: 91/972	A dedicated thalassemia hospital located in Dhaka (BTF)	[7]
HBV Infection	Attending patients with multiple blood transfusions	2017	523	0.01	A dedicated thalassemia hospital located in Dhaka (BTF)	[7]
	Attending patients with transfusion-dependent Beta thalassemia	2017-18	148	3.37	A dedicated thalassemia hospital located in Dhaka (BTSH)	[29]
HCV Infection	Attending patients with multiple blood transfusions	2017	247	28.3	A dedicated thalassemia hospital located in Dhaka (BTF)	[7]
	Attending patients with transfusion-dependent Beta thalassemia	2017-18	148	13.51	A dedicated thalassemia hospital located in Dhaka (BTSH)	[29]
	Attending patients with multiple blood transfusions	2015-16	320	14.7	Dhaka Shishu (Children) Hospital Thalassemia Center (DSHTC).	[80]
HIV Infection	Attending patients with multiple blood transfusions	2017		0	A dedicated thalassemia hospital located in Dhaka (BTF)	[7]
	Attending patients with transfusion-dependent Beta thalassemia	2017-18	148	0	A dedicated thalassemia hospital located in Dhaka (BTSH)	[29]

## Clinical characteristics and patient management of thalassemia in Bangladesh

Adequate information on thalassemia clinical status, mortality rate, complications and treatment outcomes is lacking in Bangladesh.

Patients with TDT usually present early in life. A hospital-based study which mainly included patients with TDT (i.e. Beta thalassemia major, Hb E-Beta thalassemia etc.) found a mean age at diagnosis of 1.6 years. Almost all the patients presented before 5 years of age, while half of the Beta thalassemia major patients were diagnosed during infancy [17]. Another study also reported similar findings [18]. A male predominance was noted in published reports. Studies reported that the most common presenting symptom was weakness or progressive pallor and the most common presenting signs were anemia and splenomegaly. Other clinical presentations included jaundice, abdominal swelling, anorexia, and palpitation [17–19]. While the patient with TDT received their first transfusion before the age of 5 years, the age of the first transfusion of NTDT patients ranged from 13 to 60 years [12]. It should also be mentioned that patients with Hb E-Beta-thalassemia show different phenotypic variability at different stages of development [34], since numerous factors have been identified to be associated with disease severity of the NTDT patients.

The standard treatment for thalassemia patients requires a multidisciplinary approach, involving specialties like hematology, transfusion medicine, endocrinology, cardiology, nutrition, and mental health services. However, most hospitals and clinics lack access to these comprehensive services in Bangladesh. As a result, most of the patients with thalassemia remain out of standard care in Bangladesh. A study conducted in one of the renowned tertiary care hospitals in Bangladesh found that a significant proportion of individuals with thalassemia have never sought consultation with a hematologist or visited a hematology outpatient department (OPD). Among these, about 10% of those with TDT, and 50% with NTDT have not received specialized hematological care [20].

Blood transfusion is essential for managing thalassemia, but ensuring safe blood remains a major challenge in Bangladesh. Two-thirds (67%) of thalassemia patients in Bangladesh are transfusion-dependent who have to receive blood regularly for as long as they live [7]. About 42% of these patients require one to four bags of blood every month [14]. The country has 201 government and 145 private blood banks, but advanced facilities like refrigerated centrifuges, pre-storage leukodepletion, NAT (Nucleic Acid Amplification Testing) and genotype-matched transfusions are scarce; these are available only in selected centers in big cities [21]. Therefore, it is easily discernible that most, if not all, patients with TDT do not get adequate and safe transfusion in Bangladesh. As a result, thalassemia patients are at risk of various blood-borne diseases. Hepatitis C virus (HCV) is the most common among those diseases. Studies have found a higher prevalence of HCV infection among multi-transfused thalassemia patients, ranging from 13 to 28%. Similarly, Hepatitis B virus (HBV) was found in around 3% of patients. HIV infection was not found among thalassemia patients in Bangladesh as the prevalence of HIV is very low already (Table 2).

Due to regular blood transfusions and the breakdown of red blood cells, thalassemia patients accumulate excess iron in their bodies, which can accumulate in vital organs such as the heart and liver, slowly rendering them dysfunctional. Uncontrolled iron overload increases the risks of serious complications like heart failure, liver cirrhosis, endocrine dysfunction, and even hepatocellular carcinoma, Therefore, regular and adequate iron chelation (removal of iron from the body) is required for long-term survival. However, around 50% of patients with TDT could not adhere to iron chelating agents in Bangladesh. Causes of non-adherence include higher costs, unavailability and complications [22]. A study among TDT patients at a tertiary care setting reported that serum ferritin levels were significantly higher in TDT patients with a mean value of more than 4000 ng/ml [23]. This value is significantly higher than the recommended value for adequate iron chelation in TDT patients (i.e. below 1000 ng/ml). It is clear that patients in our country are poorly chelated and are at risk of serious complications related to iron overload.

Advances in fetal hemoglobin (HbF) inducers like hydroxyurea, thalidomide, and luspatercept offer hope in reducing or eliminating transfusion risk in TDT patients. Luspatarcept is not available in Bangladesh. Hydroxyurea is the most commonly used HbF inducer in Bangladesh, but it has limited effects [24]. Thalidomide recently has shown promising results in reducing transfusion needs in TDT patients [25]. It is being increasingly used by clinicians in Bangladesh with good efficacy and safety [26]. Thalidomide could serve as a promising treatment option for thalassemia patients in low-income countries like Bangladesh. However, it is still not recommended by Thalassemia International Federation (TIF) for the treatment of thalassemia.

Unlike Beta thalassemia major, Hb E-Beta thalassemia is relatively manageable if proper care is provided. Not much research on the clinical aspects of Hb E-Beta thalassemia is available, as it is localized in some selected Asian countries. Studies have found that Hb E-Beta thalassemia can be managed without transfusion in many patients but it is often managed in an ill-defined and haphazard way, usually by on-demand transfusion [16]. Unpublished data from Thalassemia clinic of Dhaka Medical College Hospital showed that with proper treatment only 16% of patients with Hb E-Beta thalassemia required regular blood transfusion. Given the high prevalence and clinical diversity of Hb E-Beta thalassemia in Bangladesh, the optimal management of these patients should be sought.

According to TIF’s Global Thalassemia Review 2023, which analyzed the facilities for the prevention and treatment of thalassemia in 51 countries of the world, Bangladesh is in category “D” both in terms of prevention and treatment [5]. This means Bangladesh is very weak in both prevention programs and thalassemia management.

### Challenges faced by thalassemia patients and their families in the context of Bangladesh

#### Managing blood transfusion regularly

Thalassemia patients and their relatives often have to struggle to collect blood regularly. Our previous study on the parents of thalassemia patients revealed that the majority of them face difficulty in managing blood donors regularly (80%) and they do not get the expected support from the organized blood donor clubs/NGOs of the country (81%) [14]. There is an intense crisis of blood donors at district and city levels in Bangladesh. About 41% of blood centers at the district level have insufficient blood supply [27]. Blood is collected mainly through blood donation programs. The activities of voluntary blood donor organisations or clubs are usually limited to urban or suburban areas. It is difficult to find a willing blood donor in Bangladesh. According to the World Health Organization report, only 31% of blood is collected through voluntary blood donors in Bangladesh and the rest comes from relatives and friends of patients [27]. Bangladesh has the lowest share of voluntary blood donation in South Asia. Thailand and Sri Lanka have ensured 100% voluntary blood donation and in India the rate is now 84% [28].

The consequences of inadequate blood transfusions can be dire such as weakness, reduced growth rate, enlarged spleen, bone deformities, and even premature death. Moreover, blood stored in district-level blood banks cannot be kept for long due to lack of proper maintenance such as; insufficient storage, mismanagement and limited shelf-life. Along with proper storage, adequate screening is required to ensure that the collected blood is safe. Thalassemia patients are at risk of various blood-borne diseases due to regular blood transfusions. A study on the patients of Bangladesh Thalassemia Society Hospital showed that 13.5% and 3.4% of the thalassemia patients who came for treatment were infected with hepatitis C and hepatitis B virus respectively. Those who receive blood more frequently also have a higher rate of contracting these infectious diseases [29]. Thalassemia patients need packed red blood cells, but they are not readily available anywhere except in a few big cities such as Dhaka, Chittagong, Dinajpur, and Jessore. Repeated intake of whole blood instead of packed red blood cells causes various transfusion-related complications. It is recommended to use leucoreduced packed red blood cells for TDT to prevent transfusion-related complications, especially alloimmunization [2]. However, leucodepletion facility is not currently available in our blood banks. Leucofilter is mostly available in the capital city only. However, the cost of leucofilter is almost double from the blood itself in Bangladesh. Therefore, most of the TDT patients can not afford them.

## Availability and affordability of iron chelation drugs

There are three types of iron-chelating medications– Deferoxamine injection, Deferiprone capsules/tablets and Deferasirox tablets. All three types of drugs are available in Bangladesh, but they are not given free of charge and are not readily available everywhere. These drugs are distributed free of cost in government hospitals to registered patients in India. In Algeria, thalassemia patients receive a “Chifa” card as chronic disease patients, which allows them to get free medication from pharmacies [30]. In Pakistan, the “Sehat” card provides free healthcare to the country’s underprivileged population, which is a form of health insurance for the poorest and financially weaker sections of society [31, 32]. There is no such national policy or health insurance facility in Bangladesh.

It is important to regularly monitor liver and cardiac iron concentration to prevent iron accumulation and subsequent complications. An MRI-based approach called Ferriscan is currently the most widely accepted technique for assessment of iron overload. Unfortunately, this test is available just in one or two medical centres in Bangladesh. The cost of each test is about BDT 7000 (heart) and BDT 10,000 (liver), according to BTF (Personal communication, February 2024). Besides, this test is not available in government hospitals. More affordable MRI-based techniques, such as MRI T2* and MRI R2*, also provide reliable liver and cardiac iron assessments. However, these tests are similarly limited to a few private medical centers and remain unaffordable for most, with costs ranging from BDT 10,000 to 12,000 per test. The restricted availability and high cost of these diagnostic tools continue to pose significant challenges for patients and healthcare providers in Bangladesh.

### Inadequate treatment infrastructure

Treatment of thalassemia patients in Bangladesh is mainly centred in Dhaka city. According to the Directorate General of Health Services, out of 185 blood banks in Bangladesh, 116 are located in Dhaka district and the remaining 69 are located in other districts of Bangladesh. Although there are 666 hospitals and clinics in Dhaka district, there are only 68 hospitals and clinics in each district outside Dhaka [33]. District-level treatment facilities have not yet been advanced. There is a severe shortage of haematologists in most districts of the country. Even at the town level, the main means of survival, blood transfusion, has not yet been developed. Therefore, there is inequality in health services across the country.

TIF-affiliated treatment centres like Bangladesh Thalassemia Samity Hospital (BTSH), Lab One Foundation of Thalassemia, Bangladesh Thalassemia Foundation (BTF) are located in the capital city. These treatment centres offer daycare blood transfusion services, medicines and lab tests at subsidized prices for member patients. In addition, the haematology department of Dhaka Medical College Hospital and Dhaka Children’s Hospital, BSMMU are providing multidisciplinary care to thalassemia patients. However, compared to the need, these opportunities are very limited and almost non-existent outside the capital city. Organized treatment facility is available only in thalassemia treatment centres in Dhaka and Chittagong. About 68.3% of the country’s population lives in villages [8], making it difficult for them to regularly visit cities or the capital for treatment. Only financially capable families can provide long-term medical care for thalassemia patients. Most patients are deprived of adequate and standard treatment. The majority of the patients in Bangladesh die without proper treatment or without even knowing that they have the disease.

The tribal population in Bangladesh (living in tribal areas such as hill tracts) may face challenges in accessing healthcare services due to geographical remoteness, limited infrastructure, and cultural factors such as language barriers. This is an important issue to address in the context of thalassemia prevention, as the epidemiological figures among the tribal community are alarmingly different.

Monitoring and evaluating the health of thalassemia patients and its complications requires multidisciplinary treatment involving paediatricians, haematologists, transfusion medicine specialists, cardiologists, endocrinologists, gynaecologists, nutritionists, psychiatrists, dentists and strong blood bank infrastructure [34]. In a country like Bangladesh, these multidisciplinary facilities are usually unavailable in most government hospitals and private clinics. The government of Bangladesh published national guidelines on thalassemia care in 2019 for physicians [35], which is commendable, but the health system is yet to be developed to implement it.

### Financial burden

About 18.7% of people in Bangladesh currently live below the poverty line [36]. Bangladesh lags far behind other South Asian countries in terms of out-of-pocket health expenditure. The financial impact of thalassemia on patients and their families is immense as patients in this country have to bear a large portion (74%) of the costs of treatment, medicines, and other health services from their own pockets [37]. In India, Pakistan, Nepal and Sri Lanka this rate is between 45 and 55%.

A TDT patient is expected to spend approximately BDT 19,000 per month to receive minimal standard treatment (Table 3). Most of the families of thalassemia patients belong to the lower and middle-income class, for whom it is quite difficult to spend this much money per month. The cost of thalassemia treatment consumes a large part of the income of low or middle-income families and sometimes the treatment cost of a family is higher than the average family income [7]. As a result, the patient as well as the whole family suffers psychologically and financially. A recent study has shown that an average thalassemia patient can spend only around BDT 2,000 per month on treatment (Table 3). The large discrepancy between estimated costs and actual expenditure results in inadequate treatment. In the absence of universal health coverage and health insurance facilities at the national level, most patients survive with inadequate treatment.

**Table 3:** Monthly treatment cost per patient of thalassemia

In BDT	Presumptive cost ^a^ (Conservative estimates) ^c^ [7]	Actual expenditure ^b^ (Empirical evidence) [81]
Total	18,954	2003
Blood transfusion ^d^	7371	925
Iron chelation	10,530	1510
Lab test	1053	-
% of family income		13%

^a^ USD is converted to BDT as per November 2017 rate as USD 1 = BDT 81

^b^ Annual figures are converted to monthly

^c^ The cost varies widely depending on the patient’s age, weight and clinical condition. So, the cost for a patient aged 11–20 years is considered as average

^d^ Includes the cost of transfusion materials, procedural charges, laboratory charges for haemoglobin tests and OPD ticket charges for doctor’s consultation or hospitalisation

Since 2019, the Department of Social Services has been providing support to poor patients with cancer, kidney, liver cirrhosis, paralysis by stroke, congenital heart disease and thalassemia with BDT 50,000 per person per year [38]. This grant is not universal; the approval is subject to the application verification, the candidate’s eligibility and budget availability. As there is no facility for free medicine or lab tests for thalassemia in the country, this budget is inadequate given the large number of thalassemia patients and unaffordable treatment costs. Although thalassemia patients are included in the definition of disability in India, Canada and some other countries, they are not included in the Disability Act of Bangladesh yet. Therefore, patients are not eligible for disability benefits such as disability allowance, stipend, job quota, interest-free loans, or reserved seats in public transport.

### Psychosocial challenges

Thalassemia patients do not usually want to disclose their disease for fear of social rejection or discrimination, as reported by a qualitative study from Iran [39]. Our study found that their fear is reasonable as our previous study in Bangladesh found that 17.3% of college students in Bangladesh do not want to be friends with thalassemia patients and 14.4% do not want to donate blood to thalassemia patients [40]. Families of thalassemia patients participate less in social events and feel socially isolated, according to a study from Thailand [41]. Similarly, our study found that more than 40% of the patients’ parents face social stigmatisation in Bangladesh [14]. Due to the lack of awareness, misinformation and superstition, thalassemia is considered a ‘blood fault’ that isolates patients’ families socially. Our unpublished (under review) study confirms that almost half (46.8%) of the mothers having children with thalassemia agree to the fact that their social life is hampered [42]. Studies in this aspect are still scarce in Bangladesh.

Physical adversity also affects the mental health of patients, but this issue remains neglected due to the preoccupation with dealing with the disease. Mental health takes a huge toll on patients as well as their families. Mothers, as the primary caregivers, suffer from anxiety (~ 58%), depression (~ 63%), and stress (~ 62%) in Bangladesh [42]. The underlying cause of social and emotional problems is their concern with the desire to live a long and quality life. As the number of adult patients increases as a result of advances in medical science, concerns about their quality of life and mental health are increasing. With increasing age, disease-related complications may also rise. Patients are always worried about the uncertainty of receiving blood regularly. Bone deformity, changes in facial structure and disrupted growth are common consequences of inadequate treatment. Because of these, some thalassemia patients—talented graduates—are refused to be hired by employers. Starting a family is also very uncertain for them. Our unpublished (under review) study found that 90.4% of mothers are concerned about their thalassemic child’s future (education, job, marriage) [42]. Adult patients are realising at some point that adequate and regular maintenance of proper treatment can lead to better survival. Patients from developed countries are now encouraged to lead a normal life, pursue higher education, work in a job, get married and have children [43]. However, the patients in Bangladesh are lagging in this regard. If adequate support is provided systematically in specialized treatment centres and if awareness among the mass can be improved, these things can become easier in patients’ lives.

### Expensive curative treatments

Blood transfusion and iron chelation are not curative treatments for thalassemia. So far, the only conventional curative method for thalassemia is bone marrow transplantation, which is very expensive. Bone marrow transplantation for thalassemia is still to be established in Bangladesh. Dhaka Medical College Hospital, Evercare Hospital and Dhaka CMH (Combined Military Hospital) have performed bone marrow transplantations of cancer patients as well as a very small number (1.2%, 2 out of 163) of thalassemia patients that costs BDT 1.4 million on average [44]. Apart from the high cost, finding a bone marrow donor is a challenge. Transplant from matched family donor (sibling or parents) is only allowed in Bangladesh. Transplant form matched unrelated donor is currently unavailable in Bangladesh as there is no national bone marrow registry. Besides, some sibling refuses to donate marrow out of fear of complications even after proper counselling. On average, this procedure has a mortality risk of about 9%, while the success rate, i.e. the chance of having a thalassemia-free life, is only 83% [45]. Overall, the success of bone marrow transplantation depends on the patient’s age, bone marrow matching and the patient’s clinical condition.

Gene therapy has emerged as a promising treatment for beta thalassemia, offering potential cures, particularly for patients with transfusion-dependent beta-thalassemia. It was first approved by the US Food and Drug Administration (FDA) for beta thalassemia in 2022. Clinical trials demonstrated significant efficacy, with many patients achieving transfusion independence [46]. However, gene therapy is highly expensive and requires specialized medical infrastructure. This costly procedure, though available in developed countries, is unlikely to reach patients in Bangladesh soon, as its current cost exceeds BDT 300 million [47]. In addition, there are some remedial drugs, but their use needs more research. A recently approved modern injectable drug Luspatercept can retain hemoglobin in the blood. Its 25 mg price in India is around Rs. 41,000 (USD 491.50) (Corsantrum Technology- medicine exporter, personal communication, December 8, 2023). The injection has to be taken every three weeks, which means a patient weighing 50 kg may need to spend at least Rs. 92,000 (∼USD 1103) per month for this. All in all, the curative or supportive measures for thalassemia are inadequate, so prevention is the must.

### Awareness of thalassemia in Bangladesh

People’s ignorance is a barrier to awareness. The people in Bangladesh know very little about this disease. Bangladesh Thalassemia Samity and the Government of Bangladesh have been trying to increase thalassemia awareness by sending short messages on mobile phones for the past few years. Yet more than half of the people (53%) had never heard of thalassemia [48]. The recent national estimate regarding this is even much higher (81.6%) [10]. And despite hearing the name, there are many misconceptions about thalassemia in people’s minds. About 21% of people do not know the disease is hereditary, many do not know exactly why the disease occurs or how to diagnose it [48].

On the other hand, our study on the parents of thalassemic children found that almost none of them (97%) had heard of thalassemia before their child was diagnosed with the disease, none of them had a premarital blood test, and most (91%) of the parents regretted not having a premarital blood test [14]. Almost half of the parents who participated in that study did not know that thalassemia is preventable (51.5%) and that carriers can lead healthy lives (54.5%). Only one-third (35.6%) of parents had heard of prenatal testing for thalassemia diagnosis. Although one-third of parents were planning to have more children in the future, but only one-quarter (24.9%) of parents wanted to have a prenatal test, while the rest relied on their fate. Parents of patients can play a significant role in creating public awareness because others can realize the severity and socioeconomic difficulties of this disease through them. However, the lack of knowledge and limited understanding about thalassemia among the parents is a hindrance. To raise awareness of thalassemia, one book targeting university students, and parents has been published where career development is linked with thalassemia [49].

### Thalassemia prevention strategy and care in the context of Bangladesh: future perspectives

#### Preventing the birth of children with thalassemia

The sole aim of the Thalassemia Prevention Action Plan is to prevent the birth of children affected by the disease. There are two ways to achieve this: i) Preventing marriage between two thalassemia carriers, after knowing whether or not someone is a carrier through a blood test (Hemoglobin Electrophoresis) before marriage, known as premarital screening. Carriers carry the thalassemia gene silently and do not show any symptoms. The Hemoglobin Electrophoresis test is enough to be done once in a lifetime and only this test can diagnose thalassemia carriers. (ii) In the case of two carrier parents, the affected child’s birth can be prevented by screening the fetus for thalassemia, known as prenatal screening. Its purpose is to legally carry out an abortion on the advice of a doctor if the unborn child is diagnosed with thalassemia disease. Thus, theoretically, thalassemia is 100% preventable.

### Why is the thalassemia prevention strategy different from other diseases?

Thalassemia is usually confined to malaria-prone areas of the world and does not spread like an infectious disease. The issue of thalassemia is localized in the global thalassemia belt (thalassemia-prone regions). Thalassemia is neglected in international research because thalassemia is not a major health concern in developed countries. Therefore, the lack of international funding and cooperation appears to be the major setback to tackling this preventable disease. Funding and patronage of international health agencies and major NGOs are at the heart of Bangladesh’s success in children’s vaccination and preventing various infectious diseases (such as diarrhoea, tuberculosis and worms). Despite the higher prevalence of thalassemia compared to some infectious diseases, thalassemia is not well studied. Consequently, its economic and social impact remains unknown.

A study in Sri Lanka found that 5% of the country’s health budget is required to manage thalassemia patients [16]. Another study conducted in Israel showed that prevention is at least four times less expensive than treatment of thalassemia [50]. A study in Iran also found that the cost of treating a thalassemia patient per month is more than four times the cost of preventing the birth of one thalassemia child through screening [51]. Hence prevention is the most effective strategy to reduce the burden of thalassemia in a country like Bangladesh.

### Why is it challenging to implement the prevention model of other countries in Bangladesh?

Prevention programs are mainly focused on mandatory premarital screening and prenatal screening, as well as optional abortion which is a sensitive religious issue in conservative Muslim countries. Thalassemia-prone countries such as Cyprus, Greece, and Italy have succeeded in reducing the birth rate of thalassemia children by implementing simple prevention strategies such as premarital blood tests to detect carriers and prenatal fetal testing to prevent the birth of thalassemia-affected children. For example, the successful implementation of the prevention program has been possible in Cyprus due to the small population of the country and the support from the developed countries of Europe. However, studies in Muslim-majority countries such as Saudi Arabia and Iran have shown that despite mandatory premarital blood tests and genetic counselling, many carrier-to-carrier marriages could not be prevented (Table 4).

**Table 4:** Lessons learned from thalassemia prevention strategies around the world

Country	Population	Intervention strategy	Current status (Gap or Success? )
Iran	88,550,570	Premarital screening has been mandatory since 1996, and prenatal diagnosis and genetic counselling have been mandatory since 1999 [52]. If the unborn fetus is affected by thalassemia, abortion is legally performed on medical advice. Abortion is optional and insurance companies cover a large portion of abortion costs [82].	Among carrier couples, only 15–20% cancelled the marriage. The reasons are mutual feelings, family pressure, social and religious beliefs, and testing at the wrong time (just before marriage after the marriage is fixed) [982]. 26.97 births of patients with thalassemia major were prevented by screening techniques [51].
Saudi Arabia	36,408,820	Premarital screening has been mandatory since 2004 [72]. Carrier couples are advised to visit a genetic counselling clinic where a designated physician (usually a paediatrician) provides genetic counselling [53].	About 90% of high-risk (carrier-carrier) couples marry, despite knowing that their child is at risk of having thalassemia or sickle cell anaemia [53]. About 52% of college students never heard of thalassemia [83].
Turkey	82,003,882	Premarital screening has been mandatory since 2003 and is free. The name of this program is the Hemoglobinopathy Prevention Program. This includes carrier detection, public awareness and education, screening and genetic counselling, and follow-up. Written regulations, education and prevention campaigns have been taken to prevent thalassemia since 2000 [84].	Premarital screening rates increased from 30–86% within ten years from 2003 to 2013. Children born with thalassemia are reduced by 90%. The number of newborns with thalassemias and hemoglobinopathies dropped from 272 in 2002 to 25 in 2010 [85].
Cyprus	875,899	In 1980, premarital screening was made compulsory by law. Then in 1984, prenatal diagnosis program started. Prenatal diagnosis is strongly advised for those intending to marry despite both being carriers [43]	Before the implementation of Thalassemia Prevention Program, 18–20 thalassemia children were born per year. But between 1991 and 2001, only 5 thalassemia children were born, one in every 2–3 years. No more thalassemia children were born after that [43].
Greece	10,724,599	Voluntary and free screening tests have been performed before marriage or childbearing since 1974. Prenatal diagnosis began in 1977 that identifies affected fetuses after conception to abort them [86, 87].	Before the prevention program, approximately 150–200 thalassemia children affected children with hemoglobinopathies were born each year on average [88]. With the implementation of the program, the country reached a reduction of 90% in new affected births per year in 15 years (2001-15) [89].
Sardinia, Italy	1,639,591	The prevention campaign began in 1977, involving extensive educational programs and voluntary screening for carriers using hematological and molecular tests. Genetic counselling is provided to couples identified as carriers, with options for prenatal diagnosis. Screening, counselling and prenatal diagnosis are free of charge [90, 91]. Pre-implantation genetic diagnosis (PGD: a way to select healthy embryos in advance), offered for those who do not wish to abort, started in 2002. PGD was prohibited in 2004 by law [92]. The law was changed to legalize PGD in 2014 and then PGD restarted [93].	In 98% of cases, mothers decide to abort the affected fetuses. The number of children with thalassemia has reduced from 120 to 3–5 [93]. Providing preimplantation genetic diagnosis to infertile couples as well as fertile women increased conception success rates from 11–31% [94].

Among Muslim countries, Iran has been emphasizing the implementation of thalassemia prevention measures for 20 years [52]. As part of the program, Iran has legalized abortion for fetuses with thalassemia and initiated free prenatal blood tests and prenatal screening facilities. Despite such efforts, Iran has not achieved the expected success in eradicating thalassemia. A closer look reveals that there is less real awareness of thalassemia in Iran. The same scenario exists in Saudi Arabia. Even though blood tests before marriage are mandatory, more than half of college students (including married couples) in Saudi Arabia have never heard of thalassemia. Even though the bride and groom were carriers of thalassemia, they continued the marriage in 90% of cases [53]. Since therapeutic abortion is a religiously sensitive and controversial issue, prenatal screening in other Middle Eastern countries, including Saudi Arabia, is not included in the thalassemia prevention strategy.

From the lessons learned from other countries, it appears that thalassemia prevention programs fail to achieve the desired success due to the lack of awareness. Compared to the countries that have implemented thalassemia prevention programs, Bangladesh has a huge population (more than 160 million) and limited healthcare resources i.e. screening and treatment centres. Most importantly, most people in the country are not aware of this disease.

Premarital blood tests and fetal testing facilities are also available in Bangladesh but on a limited scale. Policymakers are giving special emphasis on mandatory blood tests before marriage to prevent thalassemia. However, the Hemoglobin Electrophoresis test before marriage is not mandatory currently in Bangladesh, not even free. Aspirants have to take this test on their own initiative and with their own pocket money, which is not done due to a lack of awareness. Again, if genetic counselling is not given to both spouses in addition to the blood test, it may not prevent marriage between at-risk couples. For those carrier couples who cannot back out of marriage due to mutual emotions or family pressure, prenatal diagnosis can be a good option. According to Islamic fatwa, abortion within a certain period of pregnancy (120 days) is permissible considering the health of the mother and the physical anomalies of the fetus [54]. However, as the issue is sensitive, it needs extensive awareness to establish it in a Muslim-majority country like Bangladesh.

### High school-centric intervention should be the cornerstone of the thalassemia prevention strategy

It has been demonstrated by earlier research conducted in other countries that screening and teaching about thalassaemia should be directed at high school students (age 16 and up) to prevent the birth of affected children [55, 56]. According to the Bangladesh Bureau of Statistics, girls are married on average at the age of 19 in rural areas and at the age of 20 in urban areas [8]. Due to the success of various government programs (including stipends) for the expansion of women’s education, more than 80% of females study up to the secondary level [57].

**Fig. 2:**
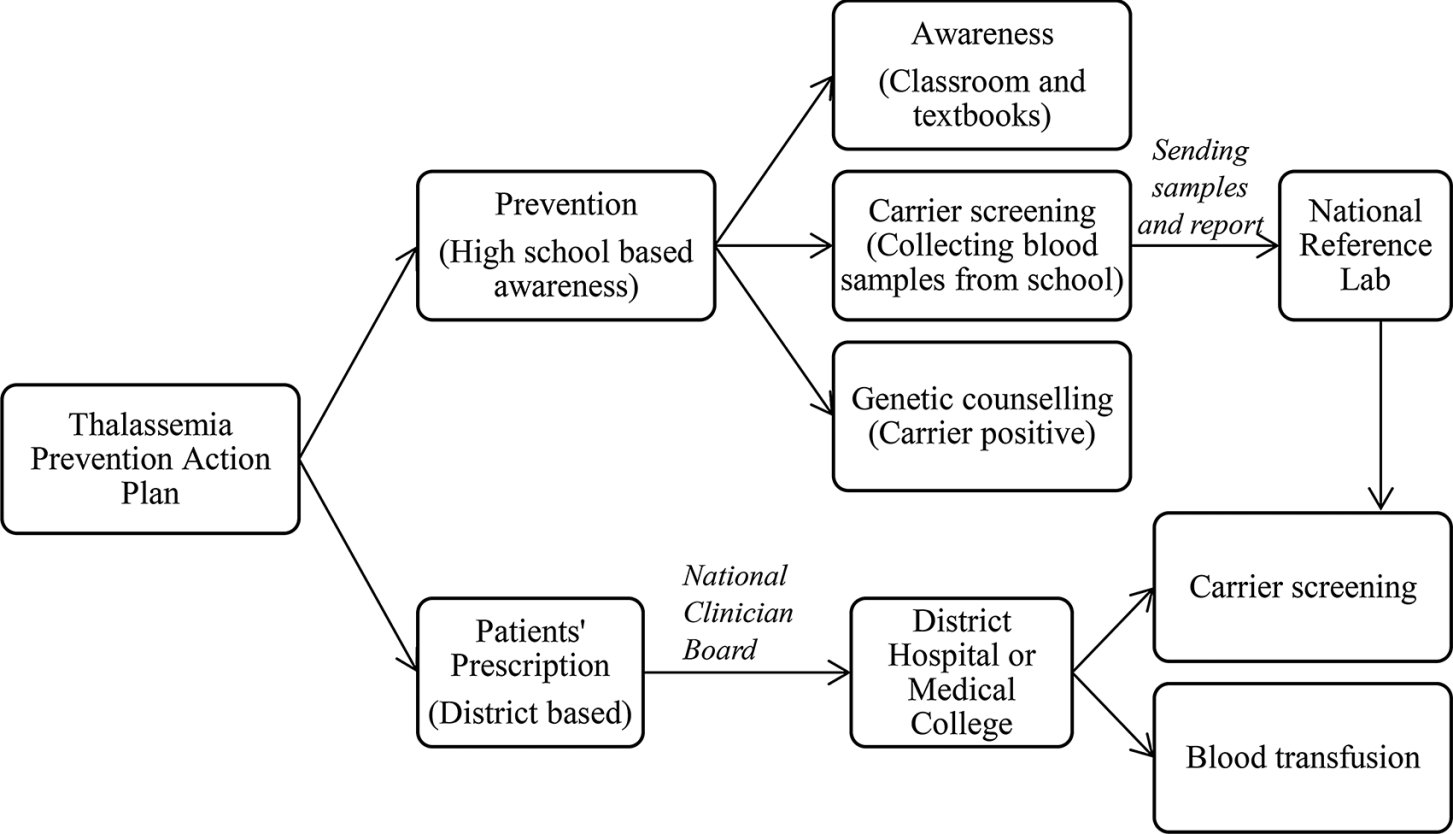
Thalassemia Prevention Action Plan in the Context of Bangladesh

High school student-centred awareness programs, screening, genetic counselling, and district-based medical care facilities should be key to the Thalassemia Prevention Program in Bangladesh (Fig. 2). A long-term action plan based on the country’s established system (in health and education) and untapped resources in the community should be implemented step by step to succeed in the prevention of and clinical management of thalassemia.

Teachers are highly respected in society and students at the high school level are generally obedient to their teachers. Therefore, teachers can play a key role in creating effective awareness about the dangers and prevention of this disease through textbooks and classroom discussions. The cost of this prevention strategy will be much lower. Media-based awareness alone is less likely to be effective in the context of Bangladesh, as it is a different kind of disease, where social issues are involved.

Various school-based health interventions and projects in Bangladesh have already been quite successful; such as hygiene education [58], menstrual awareness [59], mental health [60], deworming [61] etc. The reasons for these successes are the facility of using the existing institutional system of the school, the special respect of teachers in the society and the importance of their words to the general public and the high enrollment rate of boys and girls at the school level. Such a school-based project on the prevention of thalassemia should be planned.

Incidentally, our study on 1578 college students found that about 67% of students had not heard of thalassemia before although the government’s Directorate General of Health sent text messages to all mobile phone users about thalassemia as part of awareness initiatives around a month before our survey [40]. Of those who heard about thalassemia, 82% were studying science, 16% arts, and 22% commerce. The ninth-grade biology course in the science department sheds some light on thalassemia, which is why they heard the name. Only 33% of secondary school students and 21% of higher secondary students in the country study science, which is mainly city-centric [57]. On the other hand, we already discussed that most people who know the name of the disease have a misconception about thalassemia. The prevention program should be targeted at the large share of the country’s population who live in rural areas (about 68.5% according to the latest census).

Many people, even after learning about the disease, may delay screening for carrier diagnosis. Regular motivation and supervision of class teachers can be effective in implementing awareness in screening. To create long-term awareness, the topic of thalassemia needs to be included in the syllabus of all departments of ninth and tenth classes (science, arts, commerce). The government has formulated ‘National Strategy Paper 2017-30’ for adolescent health and other services. The continuity of this work is important. The issue of thalassemia awareness can be included in this program.

Carriers of thalassemia are not sick; they live a healthy life in most of the cases. However, carriers are also thought to be ill due to misinformation and socially negative perceptions about the disease, which was found in our previous research. Nearly two-thirds (64.5%) of the college students who had heard of thalassaemia were not aware of the fact that thalassaemia carriers are essentially as healthy as non-carriers [40]. Therefore, if genetic counselling is not included in the program after identifying the carriers, adverse situations will be created in the community. School teachers can play a role in genetic counselling under the supervision of experts.

### Building treatment centres for patients

In 2018, the Ministry of Health undertook free prenatal testing of thalassemia carrier-risk couples and a national registry of thalassemia patients [62]. The target was set to eliminate thalassemia from Bangladesh by 2028. But there is still no initiative in this regard and there is no national-level data on patient mortality, age-wise distribution and financial burden. BTSH and BTF are two well-known thalassemia treatment centres located in Dhaka, each of them has about 4500 registered patients. But these are only centre-based internal registrations. These must become national registration programs with government support. Registration of all thalassemia patients at the national level will facilitate the participation of thalassemia patients in all fields, including employment. A health card or insurance should be arranged for Thalassemia patients like Pakistan’s “Sehat Card” or Algeria’s “Chifa Card”.

Several discussions are organized on International Thalassemia Day every year in Bangladesh as well, but these discussions are mostly limited to preventive measures. It is time to focus on improving the quality of life of existing patients along with prevention. Patients need to be assured of proper medical care, blood transfusion and the chelation of excess iron deposited in the body should be addressed. Thalassemia patients are also benefiting from medical advances, reducing their mortality and increasing their quality of life. Earlier, Thalassemia patients used to die at a very young age due to lack of adequate treatment. With increased awareness, many patients can now lead fairly normal lives with regular treatment, but they need more support to continue this fight. Thalassemia is still thought by many to be a disease of children. Due to the increase in the average life expectancy of patients, there are many elderly patients now, they should not be forgotten, and their participation in all aspects of society and their normal life should be ensured.

According to the Directorate General of Health Services, the government will soon set up 8 thalassemia treatment centers across the country, two DNA test labs in Dhaka and Chittagong, and provide free blood, transfusion equipment and iron chelation drugs to patients [35]. Speedy implementation of these announcements is needed. Given our economic condition and limited resources, it seems reasonable to build at least one district-based patient support system where trained doctors, healthcare providers, safe blood and adequate medicines at affordable prices should be ensured. National guidelines for the treatment of thalassemia [35] should be ensured to reach the hands of doctors treating thalassemia patients in all parts of the country and training should be provided for them. A national expert committee can be formed to fill the shortage of hematologists at the district level, under whose supervision pediatricians can provide medical services in the districts.

Although a thalassemia carrier screening center could not be set up in every district in the first phase, a pipeline can be set up to allow blood samples to be tested at a National Reference Lab in Dhaka. There is a serious crisis of blood in the country, especially at the community level. Modernization of existing blood banks for blood preservation along with the creation of a community-based blood donor database can be helpful in this regard. Joint efforts of private and public will accelerate the blood collection initiative and also create awareness about thalassemia. Emphasis should be given to developing a step-by-step implementation of thalassemia screening, genetic counselling and treatment centers at district hospitals or medical colleges.

### Limitations

This review is limited by the reliance on peer-reviewed and English-language sources, potentially excluding relevant studies in other languages or formats. The data mainly focuses on specific populations, which may not fully reflect the diversity across all regions of Bangladesh. The lack of longitudinal studies on clinical outcomes and prevention program effectiveness restricts long-term impact assessment. There is also insufficient data on healthcare infrastructure, particularly on the distribution of treatment centers and specialized clinics. While social and cultural barriers are acknowledged, the review does not deeply explore region-specific factors or provide a comprehensive sociocultural analysis. Additionally, the focus on prevention strategies leaves less emphasis on the ongoing treatment and management challenges for existing patients, especially in rural areas. Lastly, while international models are referenced, their direct applicability to Bangladesh’s unique context is not fully examined.

## Conclusions

Thalassemia remains a major public health challenge in Bangladesh, with high carrier prevalence and significant socio-economic burdens due to inadequate treatment infrastructure and access to care. Despite global advances, the country struggles with limited resources, including safe blood transfusion services, iron chelation therapy, and multidisciplinary care.

This review highlights the urgent need for a comprehensive prevention strategy tailored to Bangladesh’s socio-economic context. By implementing high school-based education, carrier screening, and genetic counseling, the country can better address thalassemia. Strengthening public awareness, establishing district-level treatment centers, and ensuring affordable healthcare will be key to improving care and reducing the disease’s burden. Thalassemia is preventable through premarital and prenatal screening, and prioritizing prevention can significantly reduce its impact. This review provides a roadmap for policymakers and healthcare providers to implement effective strategies for prevention and care.

## Electronic supplementary material

Below is the link to the electronic supplementary material.

Supplementary Material 1

## Data Availability

All data generated or analysed during this study are included in this article and its additional files.

## Abbreviations

BDT: Bangladeshi Taka
BRF: Biomedical Research Foundation
BSMMU: Bangabandhu Sheikh Mujib Medical University
BTF: Bangladesh Thalassemia Foundation
BTSH: Bangladesh Thalassemia Samity Hospital
CMH: Combined Military Hospital
DNA: Deoxyribonucleic Acid
HBV: Hepatitis B virus
HCV: Hepatitis C virus
HIV: Human Immunodeficiency Virus
MRI: Magnetic Resonance Imaging
NAT: Nucleic Acid Amplification Testing
NGO: Non-Governmental Organization
NTDT: Non-transfusion-dependent thalassemia
TDT: Transfusion-dependent thalassemia
TIF: Thalassaemia International Federation
USD: United States Dollar
